# Association Between Steady-State Lactate Dehydrogenase Levels and Sickle Cell Complications: A Systematic Review

**DOI:** 10.1055/s-0045-1811706

**Published:** 2025-09-26

**Authors:** Sagad O.O. Mohamed, Amged Mohammed, Fatima S.K. Salih, Hozifa A.M. Elgadal, Ayman A.A. Elsamany, Mohamed S.K. Salih, Huda M.A. Mustafa, Israa Elawad, Mona G.A. Ahmedkaroum, Safaa G.A. Saeed, Rowa E.A. Ibrahim, Esraa A. Mohamedien, Aya A.H. Babiker, Esraa T. Suliman, Eman O.E. Mohamed

**Affiliations:** 1 Department of pediatrics and child health, University of Khartoum, Khartoum, Sudan; 2 Médecins Sans Frontières, Switzerland; 3 Department of pediatrics and child health, University of Bahri, Sudan; 4 Department of Medicine, Ibri Regional Hospital, Oman; 5 Department of Medicine, Harlem Hospital Center, New York, United States; 6 Department of Medicine, Cairo University, Egypt; 7 Department of pediatrics, Sidra Medicine, Qatar; 8 Department of Medicine, University of Medical Sciences and Technology, Sudan; 9 Department of Medicine, AL-Neelain University, Sudan

**Keywords:** sickle cell disease, complications, lactate dehydrogenase, systematic review

## Abstract

Sickle cell disease (SCD) is a hereditary hemoglobin disorder characterized by vaso-occlusion and chronic hemolysis, leading to severe complications. Finding cost-effective and reliable biomarkers for predicting disease severity and identifying high-risk patients remains challenging, especially in resource-limited settings. This systematic review evaluates the association between lactate dehydrogenase (LDH) levels measured during clinical steady-state and various complications of SCD to assess its prognostic value. A systematic literature search, adhering to PRISMA guidelines, was conducted across Medline/PubMed, Web of Science, Embase, and ScienceDirect. Eligible studies included all observational studies examining the relationship between LDH levels and subsequent SCD complications. The statistical analyses were performed to calculate the pooled standardized mean difference (SMD) and its 95% confidence interval (CI). A total of 34 studies were included, highlighting significant associations between elevated LDH levels and various SCD-related complications. These included pulmonary arterial hypertension (SMD = 0.454, 95% CI: 0.032–0.875, *p* = 0.035), stroke risk through transcranial Doppler velocities (SMD = 0.651, 95% CI: 0.459–0.843, *p* < 0.001), and kidney involvement (SMD = 0.399, 95% CI: 0.014–0.785, *p* = 0.042). This systematic review reveals a consistent association between elevated steady-state LDH levels and major complications of SCD. The findings suggest a potential role for LDH as a readily available biomarker for SCD severity, underlining its potential for inclusion in clinical assessments of SCD severity, risk stratification, and tailored interventions for high-risk patients.

## Background

Sickle cell disease (SCD) is a group of inherited disorders affecting hemoglobin, primarily characterized by the production of hemoglobin S, which leads to diverse clinical manifestations. This condition affects millions worldwide, particularly those of African, Mediterranean, Middle Eastern, and South Asian descent. 1 2 3 In SCD, red blood cell sickling and hemolysis result in vaso-occlusion and subsequent ischemia. The disease is marked by acute manifestations, including repeated episodes of severe acute pain and acute chest syndrome (ACS). It also leads to chronic complications, including stroke, nephropathy, retinopathy, avascular necrosis, and leg ulcers. The severity of morbidity, frequency of crises, degree of anemia, and affected organ systems can vary significantly from person to person. 1 4 5

Despite the high prevalence of SCD, especially in sub-Saharan Africa and other regions with limited healthcare resources, identifying reliable and feasible biomarkers to assess disease severity and predict complications remains challenging. 5 Among potential biomarkers, lactate dehydrogenase (LDH) holds promise due to its role in reflecting hemolytic activity and the intensity of tissue damage central to SCD pathology. 6 7 8 LDH is an enzyme in the glycolytic pathway that facilitates the conversion of pyruvate to lactate while converting NADH to NAD + . It is widely distributed across various tissues and exists in serum as five distinct isoenzymes, which are usually included in routine serum LDH determination. 6

Elevated LDH levels are observed in SCD patients and may correlate with severity and long-term complications, making it a potential indicator of disease severity even during steady-state periods, when patients are free from acute episodes. 6 8 9 Among markers of hemolysis, LDH stands out as a valuable predictor of SCD severity due to its sensitivity, relative specificity, wide availability, and cost-effectiveness. 10 11 These factors make LDH a more practical and feasible choice for routine clinical use, particularly in resource-limited settings where accessible and cost-effective diagnostic tools are needed. Studying LDH levels during steady-state periods enables the establishment of a stable baseline assessment of chronic disease processes. In the context of SCD, “steady state” refers to a period when the patient is relatively stable and not experiencing acute complications, such as pain crises, acute illnesses, and recent blood transfusions. 1 5

Although LDH is a known marker of hemolysis, research shows inconsistent results regarding its correlation with specific complications of SCD. This systematic review aims to evaluate existing evidence on the correlation between steady-state LDH levels and severe SCD complications. Such an evaluation could strengthen the evidence supporting LDH as a prognostic marker in SCD, informing preventive interventions and improving clinical decision-making and patient outcomes.

## Methods

### Search Approach and Studies Inclusion Criteria

This review was done based on the Preferred Reporting Items for Systematic Reviews and Meta-Analyses (PRISMA) guidelines. 12 The review's protocol was registered previously on the Open Science Framework platform ( https://osf.io/gvaeb ). Search terms used for this review were (sickle cell *or* hemoglobin S disease *or* hemoglobin S disorder) *and* (lactate dehydrogenase *or* LDH). To gather relevant literature, we conducted a systematic literature search using the electronic databases of PubMed, Web of Science, Embase, and ScienceDirect. There were no restrictions applied to the search in terms of age, race, geographical area, or publication date. We reviewed the articles referenced by the included articles to ensure no possible relevant articles were missed. The publications were uploaded to the EndNote software to expedite initial screening of titles and abstracts and remove duplicate entries.

### Inclusion and Exclusion Criteria

The selection process involved a two-step approach. Initially, we screened the titles and abstracts of all identified articles to identify potentially relevant studies. Subsequently, we conducted a comprehensive full-text review of these selected studies to assess their eligibility based on the predefined inclusion criteria. Eligible studies included cross-sectional, case–control, or cohort designs that specifically reported on the relationship between serum LDH levels and complications of SCD. Eligible studies were required to clearly state that the SCD patients were in a steady state, meaning they were free from any acute conditions like acute illnesses, painful crises, or recent blood transfusions. Publications such as case reports, editorials, reviews, abstracts, or those lacking sufficient data on the relevant variables were excluded.

### Quality Assessment and Data Extraction

To evaluate the methodological rigor and potential biases in the included studies, we employed the critical appraisal checklists provided by the Joanna Briggs Institute ( https://jbi.global/critical-appraisal-tools ). The tool facilitates assessment of the possibility of bias in study design, conduct, and data analysis.

The data extraction process involved four independent reviewers for extracting relevant information from each study. Any discrepancies or inconsistencies among the reviewers were resolved through discussion and consensus. The data extracted from each study included author, year, region, number of patients, age group of the participants, and a brief summary of study findings related to LDH levels among SCD patients with and without complications. All inconsistencies during quality assessment and data extraction were resolved by discussion and consensus.

### Statistical Analysis

The statistical analyses were performed by using the Jamovi software ( https://www.jamovi.org ) to calculate the pooled standardized mean difference (SMD) and its 95% confidence intervals (CIs). The random effects model, using the DerSimonian–Laird method, was chosen to account for the high heterogeneity among studies, which we evaluated using the *I* ^2^ statistic. To test for the presence of publication bias, we performed statistical analyses using both Begg's and Mazumdar's rank correlation test and Egger's regression test. In addition, funnel plots were visually checked to evaluate publication bias for analyses involving more than 10 studies. The Duval and Tweedie trim-and-fill method was applied to account for potentially missing studies when there is evidence of a publication bias. The significance level for all analyses was set at 0.05.

## Results

### Studies Characteristics

**Fig. 1:**
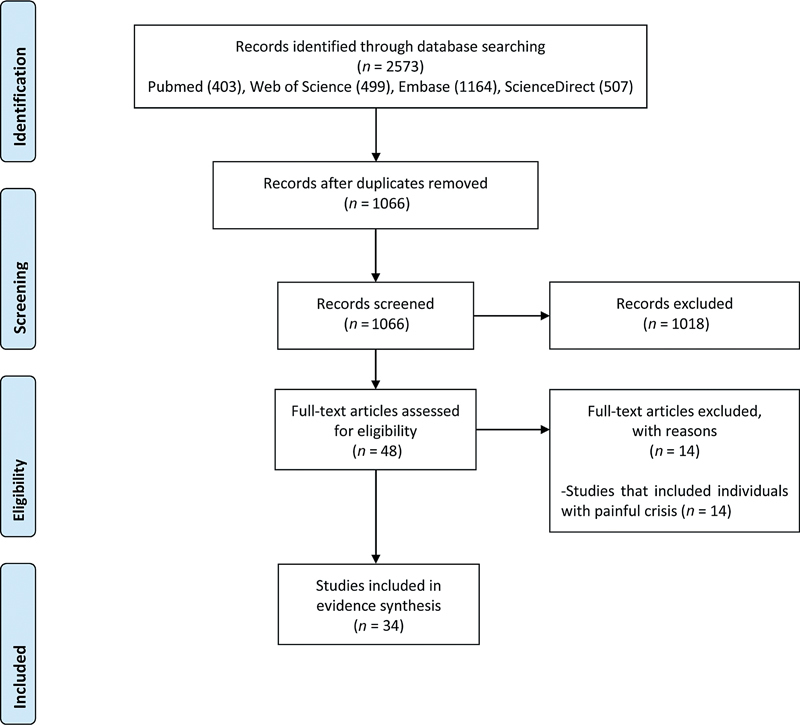
Flow chart for the study selection process.

The schematic flow of the study identification and selection process is presented in Fig. 1 . Initially, the search yielded a total of 2,573 records. After removing duplicate data, 1,066 studies were included for the title and abstract screening. Of which, 1,018 were excluded due to irrelevance. Full texts of the remaining 48 records were screened with a subsequent exclusion of 14 records, as shown in Fig. 1 . Lastly, a total of 34 studies met the eligibility criteria and were further included in this review. These studies were included in the meta-analyses.

The main features of the included studies, including risk of bias assessment, are presented in Table 1 . Sample sizes ranged from 36 to 535 participants across studies. Of them, 15 studies sampled only children with SCD, while the other studies included adult patients with SCD. Key findings of the relationship between LDH levels and the severity of complications in SCD are summarized as follows.

**Table 1:** Baseline characteristics of the studies included in the review

Study	Year	Country	No. of SCD patients	Age group	Main findings related to the correlation between LDH levels and SCD complications	Quality assessment
Adanho et al	2022	Brazil	159	<18 y	Correlation with abnormal TCD velocities	6/8
Adegoke et al	2017	Brazil	36	4–11 y	Association with vitamin D deficiency	6/8
Adekile et al	2017	Kuwait	38	<18 y	No significant correlation with pulmonary function tests	8/8
Akgül et al	2007	Türkiye	87	All groups	Association with high pulmonary artery pressure	8/10
Al-Allawi et al	2016	Iraq	94	3–39 y	No significant correlation with TRJV	8/8
Aleluia et al	2017	Brazil	99	Not available	No significant correlation with HDL-C level	6/8
Aol et al	2024	Uganda	332	6–18 y	Children with SCD who have high LDH had a nearly 2-fold higher risk of abnormal lung function	8/8
Ataga et al	2006	US	76	Adults	LDH levels were significantly associated with pulmonary artery systolic pressure	9/11
Bernaudin et al	2007	France	373	12–18 mo	LDH is an independent predictor for TCD high velocities	8/11
Connes et al	2022	Nigeria	75	Adults	Association with recurrent leg ulcer	5/8
Cumming et al	2007	Jamaica	225	18–64 y	A 100-unit increase in LDH was associated with a 19.9% increased risk of developing chronic leg ulcers	10/11
Day et al	2012	UK	253	17–70 y	LDH was the strongest predictor of microalbuminuria among other hemolytic parameters studied	6/8
Dahoui et al	2009	Lebanon	90	2–30 y	Association with abnormal TRJV values	8/8
Dosunmu et al	2014	Nigeria	56	14–42 y	Correlation with pulmonary artery pressure	8/10
Fares et al	2020	France	78	18–61 y	Microstructural changes in the retina	6/8
Garada et al	2015	Bahrain	55	18–64 y	Association with very low BMD	6/8
Gomes et al	2023	Brazil	55	≥19 y	Association with TG/HDL-C ratio	6/8
Gurkan et al	2010	US	34	5–19 y	Association with microalbuminuria	8/8
Hamideh et al	2014	US	38	11–48 y	No significant association with albuminuria	8/8
Hamdy et al	2018	Egypt	80	<20 y	Association with vitamin D deficiency	6/8
Itokua et al	2016	DR Congo	70	2–18 y	Correlation with urinary albumin creatinine ratio	8/8
Ismail et al	2019	Nigeria	100	2–16 y	Association with abnormal TCD velocities	6/8
Liem et al	2007	US	51	10–20 y	Correlation with TRJV values	8/8
Lobo et al	2015	Brazil	123	16–60 y	Association with abnormal TRJV values	8/8
Minniti et al	2009	US	310	8–16 y	Association with abnormal TRJV values	8/8
Ojewunmi et al	2017	Nigeria	147	2–16 y	Association with abnormal TCD velocities	8/8
Roger et al	2021	France	535	19–30 y	Association with the onset of CKD stage II	10/11
Seixas et al	2010	Brazil	152	Children	Association with higher triglycerides and lower HDL-C	6/8
Senet et al	2016	France	98	30–44 y	Association with the healing of leg ulcers at week 24	6/11
Silva et al	2020	Portugal	70	3–16 y	Association with stroke risk because of the correlation with time-averaged mean velocity in the middle cerebral artery	8/8
Sokunbi et al	2017	Nigeria	175	5–18 y	No significant association with TRJV	8/8
Valente-Frossard et al	2020	Brazil	161	12.41 ± 2.76 y	Association with higher HDL-C levels	6/8
Youssry et al	2015	Egypt	47	5–19 y	Association with microalbuminuria	8/8
Zorca et al	2010	US	365	27–45 y	Association with higher triglycerides and lower HDL-C	6/8

Abbreviations: BMD, bone mineral density; CKD, chronic kidney disease; HDL-C, high-density lipoprotein cholesterol; LDH, lactate dehydrogenase; SCD, sickle cell disease; TCD, transcranial Doppler; TG, triglyceride; TRJV, tricuspid regurgitant jet velocity.

### Association between LDH and Cardiopulmonary Complications

The main cardiopulmonary complications assessed across studies were abnormal lung function and pulmonary hypertension. Regarding lung function, only two studies assessed abnormal lung function, specifically forced vital capacity and forced expiratory volume in 1 second. 13 14 One of them revealed a significant negative correlation with LDH levels. Conversely, several studies assessed the link between LDH levels and pulmonary hypertension. These studies used tricuspid regurgitant jet velocity as a surrogate marker for the condition. 13 15 16 17 18 19 20 21 22 23

**Fig. 2:**
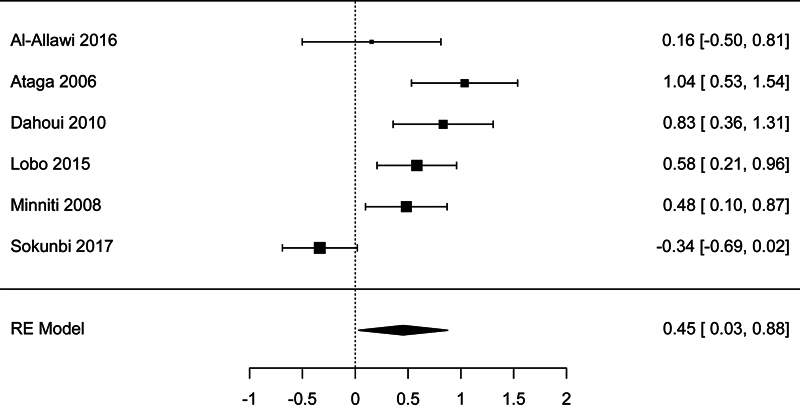
Forest plot for the analysis of the association between lactate dehydrogenase and pulmonary hypertension.

There were six studies with sufficient data to be included in the meta-analysis of the association between LDH and pulmonary hypertension. The meta-analysis showed a significant association, with a pooled effect size SMD = 0.454 (95% CI: 0.032–0.875, *p* = 0.035; Fig. 2 ). There was high studies heterogeneity according to the *I* ^2^ test (81.8%). However, there was no publication bias detected according to the Begg's test ( *p* = .469) and Egger's test ( *p* = 0.663).

### Association between LDH and Metabolic Disorders

Several studies pointed to relationships between LDH and metabolic health indicators, especially high-density lipoprotein cholesterol (HDL-C) and triglyceride levels. 24 25 26 Pereira Gomes et al found a moderate positive correlation between LDH levels and atherogenic index, triglyceride:HDL-C ratio. 27 Similarly, higher triglyceride and lower HDL-C levels were observed in association with increased LDH, suggesting the role of LDH in reflecting disruptions in lipid profiles. 24 25 28 Additionally, Adegoke et al and Hamdy et al highlighted that patients with vitamin D deficiency had significantly higher LDH levels, suggesting a possible link between LDH and nutritional deficiencies. 29 30

**Fig. 3:**
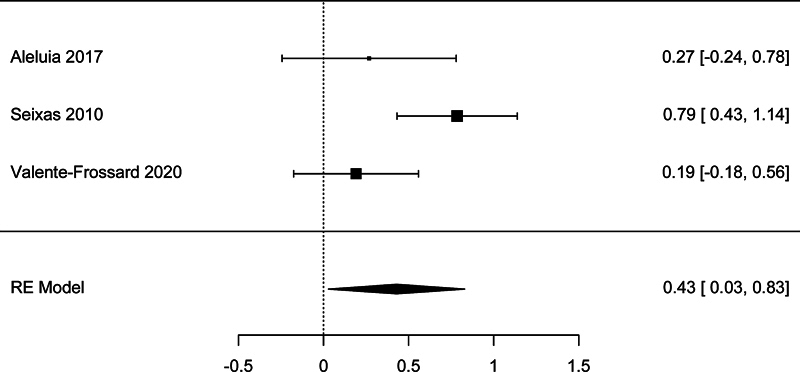
Forest plot for the analysis of the association between lactate dehydrogenase and low high-density lipoprotein cholesterol.

There were three studies with sufficient data to be included in the meta-analysis of the association between LDH and low HDL-C (<40 mg/dL). The meta-analysis showed a significant association, with a pooled effect size SMD = 0.430 (95% CI: 0.030–0.830, *p* = 0.035; Fig. 3 ). There was high studies heterogeneity according to the *I* ^2^ test (65.8%). However, there was no publication bias detected according to the Begg's test ( *p* = 1.00) and Egger's test ( *p* = 0.616).

### Association between LDH and Cerebrovascular Complications

Elevated LDH has been positively correlated with transcranial Doppler (TCD) velocities, which were used to assess cerebral blood flow velocity and predict stroke risk among SCD patients. The studies used different cut-points to define abnormal TCD velocities. However, most of them showed a significant association between LDH levels and TCD velocities. Bernaudin et al found LDH to be an independent predictor of increased TCD velocities. 31 Other studies have corroborated these findings, demonstrating positive correlations between LDH levels and TCD velocities, particularly in the middle cerebral artery. 32 33 34 35 Silva et al and Domingos et al pointed out that LDH levels were higher in patients at risk for stroke. 36 37

**Fig. 4:**
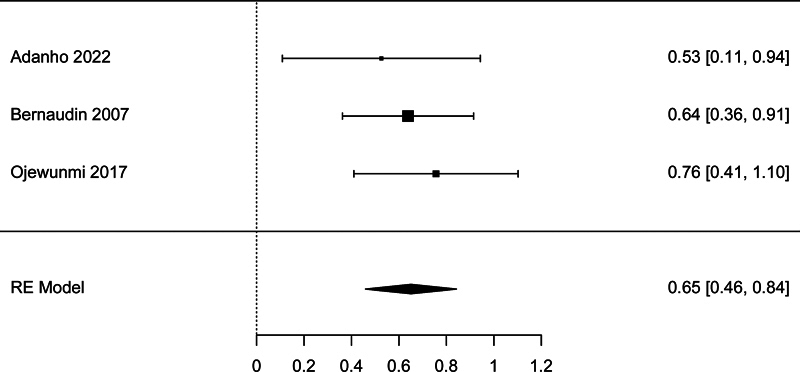
Forest plot for the analysis of the association between lactate dehydrogenase and transcranial Doppler.

There were three studies with sufficient data to be included in the meta-analysis of the association between LDH and TCD. The meta-analysis showed a significant association, with pooled effect size SMD = 0.651 (95% CI: 0.459–0.843, *p* < 0.001; Fig. 4 ). There was no heterogeneity detected according to the *I* ^2^ test (0.00%) and there was no publication bias detected according to the Begg's test ( *p* = 1.00) and Egger's test ( *p* = 0.790).

### Association between LDH and Kidney's Involvement

The associations of LDH with microalbuminuria and albuminuria were explored in five studies. 38 39 40 41 42 The studies used different cut-points to define microalbuminuria and albuminuria. However, the findings revealed that elevated LDH served as a marker for kidney involvement in SCD patients. Further supporting this connection, Roger et al found that increased LDH levels were associated with the development of chronic kidney disease. 43

**Fig. 5:**
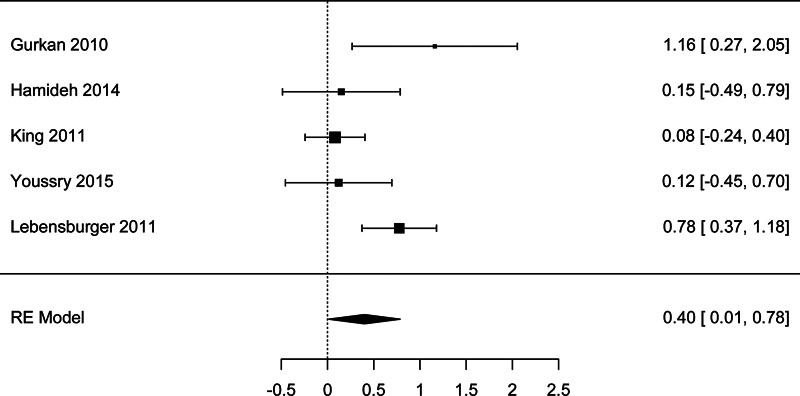
Forest plot for the analysis of the association between lactate dehydrogenase and microalbuminuria.

There were five studies with sufficient data to be included in the meta-analysis of the association between LDH and microalbuminuria. The meta-analysis showed a significant association, with a pooled effect size SMD = 0.399 (95% CI: 0.014–0.785, *p* = 0.042) ( Fig. 5 ). There was high studies heterogeneity according to the *I* ^2^ test (63.9%). However, there was no publication bias detected according to the Begg's test ( *p* = 0.483) and Egger's test ( *p* = 0.387).

### Association between LDH and Other Complications of SCD

There were three studies that demonstrated a significant relationship between high LDH levels and various manifestations of leg ulcers in SCD patients, including recurrence and poor healing outcomes. 44 45 46 Senet et al noted that lower LDH levels were associated with better outcomes in leg ulcer management. 46 Additionally, LDH levels were correlated with lower bone mineral density, suggesting a potential link between LDH and bone health in SCD. 47 Lastly, a study showed that higher LDH levels were associated with foveal avascular zone enlargement and macular ischemia, which are signs of macular vascular changes and sickle retinopathy. 48

## Discussion

This systematic review consolidated the available data and provided a more robust understanding of the association between steady-state LDH levels and several specific complications in individuals with SCD. The results support the potential of LDH as a prognostic biomarker for SCD complications because of the consistent associations across studies, which assessed LDH levels during steady-state periods, indicating its usefulness as a noninvasive marker for chronic disease assessment without interference from acute episodes.

The identified associations are explainable by the effect of the hemolysis and endothelial dysfunction, along with nitric oxide (NO) depletion. 6 26 49 Hemolysis in SCD releases cell-free hemoglobin into the bloodstream, where it rapidly scavenges NO, a potent vasodilator essential for maintaining healthy vascular function. This depletion of NO results in vasoconstriction and endothelial dysfunction, highlighting a possible connection between hemolysis and cerebral vasculopathy, likely mediated by reduced NO bioavailability. NO plays a critical role in vascular health by inhibiting smooth muscle contraction, platelet aggregation, and leukocyte adhesion to the endothelium. As hemolysis decreases NO availability, it may contribute to the development of cerebral and other forms of vasculopathy. 6 49

In SCD patients, NO depletion is linked to various vasculopathy-related complications, including pulmonary hypertension, a common condition in SCD patients. Beyond pulmonary hypertension, hemolysis and elevated LDH levels are also implicated in ACS. Hemolysis may contribute to ACS through mechanisms such as vaso-occlusion, inflammation, and potentially pulmonary thrombosis. 50 However, additional research is needed to clarify these mechanisms and assess LDH's role in predicting ACS risk.

The results on metabolic complications associated with LDH in SCD reveal significant insights.

Elevated LDH in SCD correlates with a dysregulated lipid profile, particularly marked by reduced HDL-C levels. This suggests that patients with both high LDH and low HDL-C may experience more severe hemolytic and inflammatory effects, increasing their risk for vascular complications. Elevated LDH reflects higher cell turnover due to hemolysis, which is associated with lipid profile changes, including reduced HDL-C. The decrease in HDL-C may result from oxidative stress and inflammation triggered by hemolysis, further impairing vascular health. 24

From a clinical perspective, the findings of this review have practical implications. LDH is a routine blood test, making it a readily accessible and cost-effective biomarker for monitoring SCD patients. If a strong association with complications is confirmed, steady-state LDH could be incorporated into clinical practice to identify individuals at higher risk. 8 9 51

By identifying patients with elevated steady-state LDH, clinicians could potentially implement early interventions, such as closer monitoring or adjustments to disease-modifying therapies. This could help prevent or mitigate the severity of complications. For example, because pulmonary hypertension is often asymptomatic in its early stages, regular screening with echocardiography is recommended for patients with SCD, especially those with markers like high LDH. In addition, research also revealed a relationship between LDH and creatinine clearance, pointing to its possible role in predicting kidney function decline. 52

The key limitations of this review are limitations in the available data as well as the heterogeneity of the methods used by the included studies to measure and describe data. Meta-analysis was not performed for several clinical outcomes due to significant differences in the definitions and cut-points used to define outcome measures across the included studies, which prevented quantitative synthesis of the results. The predominance of cross-sectional data makes it challenging to establish causality and introduces the risk of confounding factors that were not uniformly controlled for across studies. In addition, the search showed limitations in longitudinal studies, which would be more informative for understanding how LDH levels predict the development or progression of complications over time. Variations in LDH assay methods and laboratory-specific reference ranges likely contributed to the variability in results. Lastly, the inclusion of only English-language publications may limit the overall representativeness of the findings of this review.

## Conclusion

This systematic review demonstrated the correlation between the steady-state LDH levels and several specific complications of SCD. Steady-state LDH levels can provide valuable clinical insights into disease severity and potential complications of SCD, emphasizing its probable role for inclusion in clinical assessments of SCD severity. The findings of this review could contribute to clarifying the relationship between steady-state LDH levels and complications in SCD, help reduce disease progression, and improve patients' quality of life.

To build upon these findings and address the current limitations, future research should focus on several key areas. First, longitudinal studies are needed to track LDH changes over time in SCD patients to establish a causal link between elevated LDH and the onset or progression of complications. Second, standardizing LDH measurement is needed to reduce variability and support the development of universal risk-stratification thresholds for clinical use. Finally, future studies should investigate the utility of combining LDH with other markers of hemolysis and endothelial dysfunction to determine if a multimarker panel can improve prognostic accuracy for identifying high-risk patients.
